# Reciprocal Regulation between lncRNA ANRIL and p15 in Steroid-Induced Glaucoma

**DOI:** 10.3390/cells11091468

**Published:** 2022-04-27

**Authors:** Peixing Wan, Siyu Huang, Yanting Luo, Caibin Deng, Jiajian Zhou, Erping Long, Yehong Zhuo

**Affiliations:** 1 State Key Laboratory of Ophthalmology, Zhongshan Ophthalmic Center, Sun Yat-sen University, Guangzhou 510060, China; wanpeixing@gzzoc.com (P.W.); siyuh5565@gmail.com (S.H.); luoyanting310@gmail.com (Y.L.); dengcaibin928@gmail.com (C.D.); 2 Department of Molecular, Cellular and Developmental Biology, University of Michigan, Ann Arbor, MI 48109, USA; 3 Dermatology Hospital, Southern Medical University, Guangzhou 510091, China; zhoujj2013@smu.edu.cn; 4 Department of Ecology and Evolutionary Biology, University of Michigan, Ann Arbor, MI 48109, USA

**Keywords:** steroid, cellular senescence, glaucoma, lncRNA

## Abstract

Steroid-induced glaucoma (SIG) is the most common adverse steroid-related effect on the eyes. SIG patients can suffer from trabecular meshwork (TM) dysfunction, intraocular pressure (IOP) elevation, and irreversible vision loss. Previous studies have mainly focused on the role of extracellular matrix turnover in TM dysfunction; however, whether the cellular effects of TM cells are involved in the pathogenesis of SIG remains unclear. Here, we found that the induction of cellular senescence was associated with TM dysfunction, causing SIG in cultured cells and mouse models. Especially, we established the transcriptome landscape in the TM tissue of SIG mice via microarray screening and identified ANRIL as the most differentially expressed long non-coding RNA, with a 5.4-fold change. The expression level of ANRIL was closely related to ocular manifestations (IOP elevation, cup/disc ratio, and retinal nerve fiber layer thickness). Furthermore, p15, the molecular target of ANRIL, was significantly upregulated in SIG and was correlated with ocular manifestations in an opposite direction to ANRIL. The reciprocal regulation between ANRIL and p15 was validated using luciferase reporter assay. Through depletion in cultured cells and a mouse model, ANRIL/p15 signaling was confirmed in cellular senescence via cyclin-dependent kinase activity and, subsequently, by phosphorylation of the retinoblastoma protein. ANRIL depletion imitated the SIG phenotype, most importantly IOP elevation. ANRIL depletion-induced IOP elevation in mice can be effectively suppressed by p15 depletion. Analyses of the single-cell atlas and transcriptome dynamics of human TM tissue showed that ANRIL/p15 expression is spatially enriched in human TM cells and is correlated with TM dysfunction. Moreover, ANRIL is colocalized with a GWAS risk variant (rs944800) of glaucoma, suggesting its potential role underlying genetic susceptibility of glaucoma. Together, our findings suggested that steroid treatment promoted cellular senescence, which caused TM dysfunction, IOP elevation, and irreversible vision loss. Molecular therapy targeting the ANRIL/p15 signal exerted a protective effect against steroid treatment and shed new light on glaucoma management.

## 1. Introduction

Steroids, one of the most prescribed drugs, are mainly used in the treatment of various autoimmune and inflammatory conditions. It has been reported by National Institute on Drug Abuse (NIDA) that 1.6% of the population has been treated with steroids and this number keeps growing [1]. Despite its numerous benefits, steroid usage can cause many adverse effects on the eyes, the most important being steroid-induced glaucoma (SIG) [2,3,4].

SIG is defined as a type of open-angle glaucoma. Topical treatment with glucocorticoids introduces an intraocular pressure (IOP) increase in 30–40% of the normal population to different extents [5,6,7]. Among these steroid responders, about 3% of cases that develop will continue to have high IOP and irreversible SIG manifestations [8], i.e., optic disk cupping, attenuation of retinal nerve fiber layer (RNFL), visual field defects, and vision loss after stopping steroid treatment [9]. Considering such catastrophic visual damage and the related social burden [10], it is of great importance to investigate the molecular mechanisms underlying SIG.

Mechanistically, elevation of IOP in SIG is caused by aqueous outflow resistance at the level of the trabecular meshwork (TM). TM is a complex three-dimensional structure that consists of TM cells and an extracellular matrix (ECM) [11]. Evidence suggests that steroid-induced imbalance between the deposit and destruction of the ECM contributes to the increased outflow resistance of SIG. The deposition of ECM components includes collagen, elastin, fibronectin, and glycosaminoglycans [12,13]. In addition, degradation of ECM is blunted due to the upregulation of tissue inhibitors of metalloproteinases [14]. Notably, TM cells, another important component of TM, were involved in glaucoma pathology as well. Autophagic dysregulation [15], oxidative DNA damage [16], and fibrosis of TM cells [17] have been implicated in primary open angle glaucoma (POAG); however, whether cellular changes in TM cells are involved in SIG and its underlying mechanisms have not been investigated.

High-throughput screening technology has been widely applied in identifying genetic regulators in various pathological processes, including micro-RNAs and non-coding RNAs (lncRNA), in the context of glaucoma [18,19,20]. Inspired by this, we recruited a microarray to profile the dysregulated lncRNAs and mRNAs in TM tissues from SIG mice. LncRNA ANRIL and its target gene p15 have been prioritized as top candidates and cellular senescence showed the top enrichment scores in pathway analyses.

ANRIL is a long non-coding RNA encoded in the chromosome 9p21 region. Evidence from various studies highlighted the role of ANRIL in regulating cellular senescence under oncogenic and inflammation scenarios [21,22]. The senescence regulatory effect of ANRIL results from inhibiting its neighbor gene, p15 [23]. It has been reported that p15 can induce cell-cycle arrest, i.e., blocking the progression of cell cycle from G1 phase to S or G2/M phase. Such cross-talk between ANRIL and p15 has been consistently associated with cellular senescence in epithelial ovarian cancer [24], non-small cell lung cancer [25], and type 2 diabetes [26]. Given these clues, we hypothesized that the ANRIL/p15 signal in TM cells may contribute to SIG by regulating cellular senescence.

To test this hypothesis, we investigated the regulation and interaction between lncRNA ANRIL and the p15 gene using luciferase reporter assay and real-time PCR. The essential roles of ANRIL and p15 were tested for regulating cell cycle progression in cultured TM cells and cellular senescence in a SIG mice model. The spatial patterns of ANRIL/p15 expression were analyzed based on single-cell atlas and transcriptome dynamics of human TM tissue. The relationship between ANRIL and genetic glaucoma susceptibility was investigated by colocalization between GWAS and GTEx expression of quantitative trait loci (eQTL) signals. Our results suggested that cellular senescence of TM is the key factor in steroid-induced IOP elevation. The reciprocal regulation between ANRIL and p15 plays a major role in cellular senescence and SIG.

## 2. Results

### 2.1. Transcriptome Landscape in TM from SIG Models

C57BL/6J mice were given topical 0.1% dexamethasone phosphate (DEX) eye drops, 3 times daily for 12 weeks, to induce SIG. Mice receiving sterile PBS eyedrops and without any ocular or systematic steroid exposure (details in Methods) served as controls. IOP was measured every three days in both eyes, and the IOP of control group remained stable throughout the experiment with a mean of 12.5 mmHg. When exposed to topical DEX, IOP increased from 12.5 mmHg to 15.3 mmHg at Day 28. By the end of the 12 week treatment, the IOP of DEX-treated eyes reached 23.4 mmHg. Only the DEX-treated eyes with increased IOP (>21 mmHg) were recruited in the SIG group in the following analysis. IOP information of all the mice in this study, including the excluded mice, is presented in the Supplementary Data.

**Figure 1:**
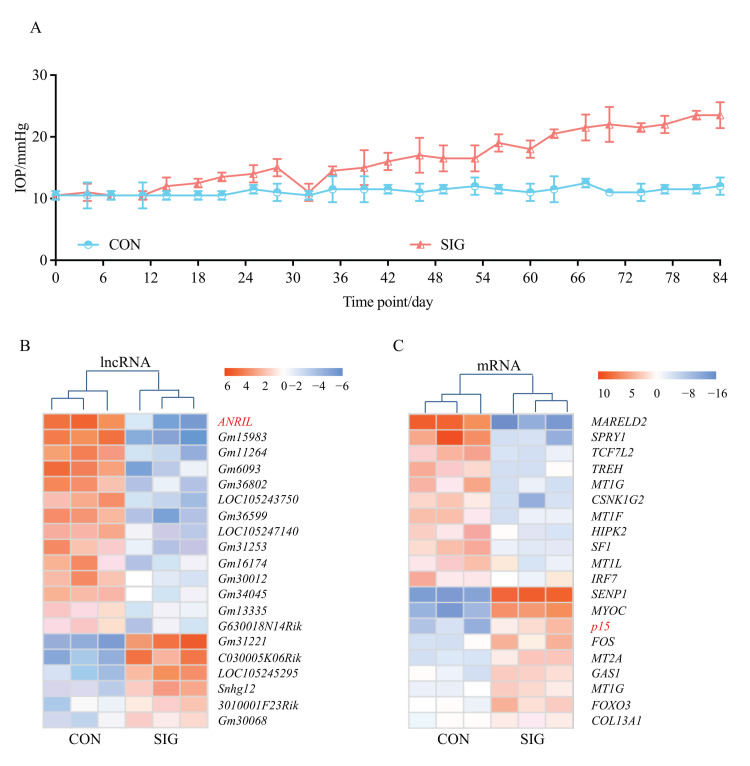
**Transcriptome landscape in SIG mice.** C57BL/6J mice were given topical 0.1% DEX or sterile PBS eye drops, 3 times daily for 12 weeks. IOP was measured in both eyes of control mice and SIG mice every three days (**A**). Only eyes with increased IOP (>21 mmHg) were recruited in the SIG group for the following microarray screening. TM tissues were isolated from SIG mice and normal controls as stated in the Methods. The dysregulated lncRNAs (**B**) and mRNAs (**C**) with a fold change >4 and *p* < 0.05 are listed in the heatmap. Notes: *n* = 3 per group; CON = control; SIG = steroid-induced glaucoma; IOP = intraocular pressure.

TM samples from three SIG mice and paired controls were collected for microarray screening (Figure 1A). A total of 527 lncRNA and 894 mRNAs were found to be aberrantly expressed in SIG. The top 20 dysregulated lncRNAs and mRNAs with a fold change >4 and *p* < 0.05 are listed in the heatmap (Figure 1B,C).

### 2.2. Correlation between ANRIL/p15 and SIG Clinical Manifestations

**Figure 2:**
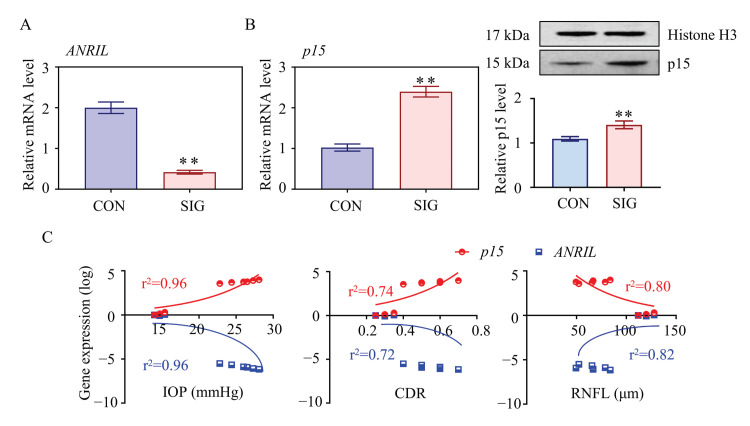
**Essential role of ANRIL/p15 in SIG progression.** The expression level of ANRIL and p15 in TM samples was measured in control and SIG groups. (**A**) lncRNA ANRIL decreased in SIG TM samples as measured by real-time PCR. (**B**) Increases in p15 were shown in SIG samples by Western blot. (**C**) The correlation between the expression level of ANRIL/p15 and ocular manifestations (IOP, CDR, and RNFL) was presented (r^2^ values were calculated from Pearson’s method, all *p* < 0.05). Notes: *n* = 5 per group; CON = control; SIG = steroid-induced glaucoma; IOP = intraocular pressure; CDR = cup disc ratio; RNFL = retinal nerve fiber layer; ** *p* < 0.01 compared with control group.

As presented in Figure 1B, ANRIL (antisense non-coding RNA in the INK4 locus) is the most differentially expressed lncRNA with a 5.4-fold change in SIG TM tissue. Decrease in ANRIL was confirmed by real-time PCR assay in SIG TM tissues (Figure 2A). ANRIL is located within the p15-p16/CDKN2A-p14/ARF gene cluster, in the antisense direction. Therefore, ANRIL could function as an antisense RNA to complement p15 mRNA, and thereby block its translation into a protein [27].

This hypothesis was confirmed by mRNA profiling of SIG TM tissues. As shown in Figure 1C, p15 was significantly increased in SIG TM compared to controls. The expression level of p15 was further examined by real-time PCR and Western Blot assay in TM tissues and was found to be increased (Figure 2B). Thus, we suspected that ANRIL/p15 interact with each other to participate in SIG development.

To define the role of ANRIL/p15 in SIG, we analyzed their correlation with steroid-induced ocular manifestations in mice. In this study, increased IOP and cup disc ratio (CDR), along with decreased thickness of RNFL was used to indicate glaucoma lesions. As shown in Figure 2C, the expression level of ANRIL is negatively correlated to IOP and CDR but is positively correlated to the thickness of RNFL. In contrast, the expression of p15 is positively correlated to IOP and CDR but is negatively correlated to RNFL thickness. With this evidence, we proposed that ANRIL and p15 are essential in SIG-induced glaucomatous manifestations with opposite effects.

### 2.3. ANRIL Is Poorly Expressed in SIG Whilst p15 Is Highly Expressed

Since ANRIL and p15 show opposite roles in the ocular manifestations (IOP, CDR, and RNFL thickness), we suspected that there might be a reciprocal regulation between ANRIL and p15 in SIG.

**Figure 3:**
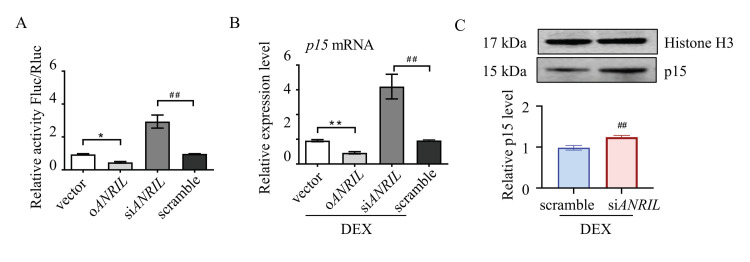
**Reciprocal regulation between ANRIL and p15.** Mice p15 gene was cloned upstream of the luciferase reporter. Mice ANRIL plasmid or siRNA were co-transfected with luciferase reporter into mouse TM cells. (**A**) The luciferase activity was greatly increased in siANRIL cells compared to scramble. In oANRIL cells, the luciferase activity was suppressed. (**B**) Depletion of ANRIL increased the mRNA level of p15 in TM cells, and overexpression of ANRIL inhibited the expression of p15, as measured by real-time PCR. (**C**) Depletion of ANRIL increased the p15 protein level in TM cells. Notes: *n* = 6 per group; oANRIL = ANRIL overexpression; siANRIL = ANRIL knock down; DEX = dexamethasone; * *p* < 0.05 compared with vector; ** *p* < 0.01 compared with vector; ^##^ *p* < 0.01 compared with scramble RNA.

To illustrate the interaction between ANRIL and p15 in TM cells, a luciferase reporter assay was used. Mouse p15 cDNA was cloned upstream of the luciferase reporter. Mouse ANRIL plasmid or small interfering RNA were co-transfected with luciferase reporter into TM cells. As indicated by the relative luminescence, luciferase activity was increased in ANRIL knock-down (siANRIL) cells compared to scramble. In ANRIL overexpressed cells (oANRIL), luciferase activity was suppressed, indicating that ANRIL blocked the transcription of the p15 gene (Figure 3A).

Real-time PCR assay was then used to test the interaction between ANRIL and p15. Our results suggested that ANRIL depletion increased the mRNA level of p15, and ANRIL overexpression inhibited the expression of p15 in DEX-treated cells (Figure 3B). We also found that knocking down ANRIL can increase the protein level of p15 in TM cells, which further confirmed reciprocal regulation between ANRIL and p15 (Figure 3C).

### 2.4. Role of ANRIL/p15 in Regulating Cellular Senescence in Response to Steroid

**Figure 4:**
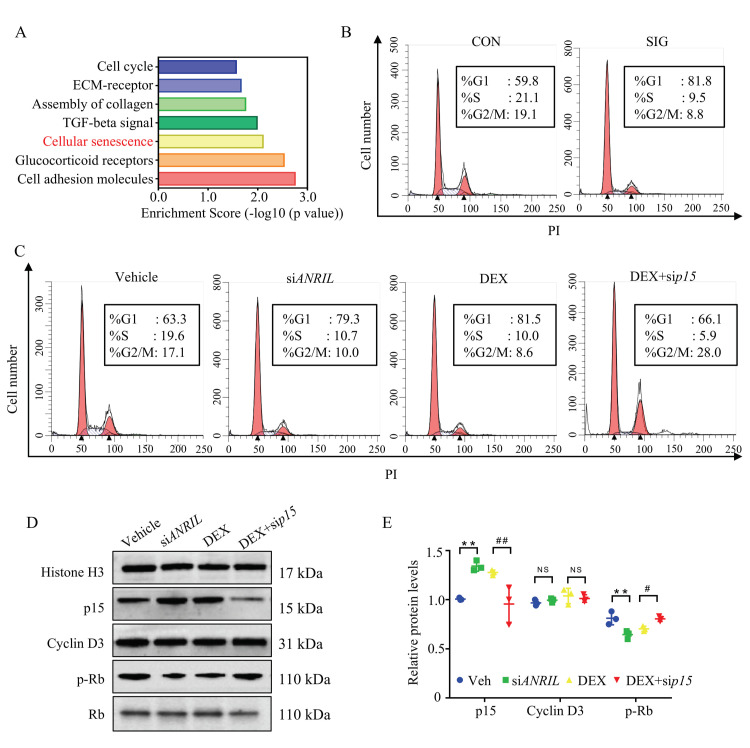
**ANRIL/p15 regulates cell senescence in SIG.** (**A**) Biological pathways participating in SIG were analyzed using Panther enrichment analyses. Disturbed genes in SIG TM samples compared to controls were enriched in the deposit of ECM and cellular senescence. *n* = 3 per group. (**B**) TM samples from control and SIG mice were prepared as single cell suspensions and labeled with PI for cell cycle analysis. In SIG TM samples, there was a huge increase of cells arrested in G1 stage and decrease in other stages. *n* = 5 per group. (**C**) In cultured mouse TM cells, DEX treatment induced G1 arrest by blocking more cells in the G1 phase compared to vehicle treated cells. ANRIL depletion also induced G1 arrest as DEX. Depletion of p15 prevented DEX induced G1 arrest. *n* = 6 per group. (**D**) In cultured mouse TM cells, the expression of p15 protein and Cyclin D3 protein was measured in nuclear protein fractions, while Histone H3 served as internal controls. The phosphorylated Rb protein was checked in cytoplasm fraction with total Rb protein as control. *n* = 6 per group. (**E**) Quantification of (**D**). Notes: CON = control; SIG = steroid-induced glaucoma; ECM = extracellular matrix; Veh = vehicle; siANRIL = ANRIL knock down; DEX = dexamethasone; sip15 = p15 knock down; ** *p* < 0.01 compared with vehicle; ^#^ *p* < 0.05 compared with scramble RNA; ^##^ *p* < 0.01 compared with scramble RNA; NS = no significant difference.

To illustrate the biological function of a dysregulated transcriptome in SIG, enrichment analyses were conducted to map the canonical pathways using Panther [28], with 6378 genes (aberrantly expressed in SIG with a fold change >2). In addition to the important role of ECM deposit, the major cellular disturbance lay in cell senescence, the permanent arrest of the cell cycle (Figure 4A). Consistently, p15 protein was reported to function as a cell growth regulator via control cell cycle G1 progression [29,30]. This evidence raised concerns about cell cycle regulation in response to steroids.

In TM samples of SIG mice, we witnessed a very large increase in cells blocked in G1 stage, and a decrease in other stages as compared to the control group (Figure 4B). Then we analyzed the cell cycle of DEX-treated mouse TM cells. Consistently, 81.5% of DEX-treated TM cells were blocked in the G1 phase compared to that of vehicle-treated cells (63.3% in G1 phase, *p* < 0.05). Our results showed that DEX treatment introduced significant G1 arrest in TM cells.

In ANRIL-depleted cells, the rate of cells blocked in the G1 stage (79.3%) was comparable to DEX treatment (81.5%, *p* > 0.05). When p15 was knocked down, DEX-induced G1 arrest could be largely rescued (Figure 4C). The percentage of G1 stage cells decreased to 66.1%. These results indicated that a loss of ANRIL in TM promoted G1 arrest by increasing p15 expression.

As reported, p15 forms a complex with Cyclin D3 to prevent the activation of Cyclin Dependent Kinase 4 (CDK4) [31]. Suppressed CDK4 kinases activity blocked proliferative cells in G1 phase. Hence, CDK4 kinase activity was recruited to illuminate the underlying mechanism of p15 in G1 arrest. Since phosphorylation of retinoblastoma protein (Rb) directly represented CDK4 kinase activity, phospho-Rb level was measured in this study [32].

As presented in immunoblot, the expression level of Cyclin D3 remained similar among the different groups (Figure 4D,E). However, the activity of Cyclin D3/CDK4 complex decreased in response to DEX treatment with compromised phosphorylation of the Rb protein (Figure 4D,E). Meanwhile, ANRIL depletion showed a similar effect as DEX treatment by downregulating the level of phosphorylated Rb protein (Figure 4D,E).

When p15 was depleted in the TM cells, the activity of Cyclin D3/CDK4 complex resumed in response to DEX, supported by the increased level of phosphorylated Rb (Figure 4D,E). The immunoblot assay results matched the cell cycle analysis in TM cells (Figure 4B,C).

In conclusion, DEX decreased ANRIL expression, and increased p15 level in TM cells. Overexpressed p15 protein suppressed Cyclin D3/CDK4 activation and Rb phosphorylation, and eventually blocked cell cycle progression.

### 2.5. ANRIL/p15 Signaling Promotes IOP Elevation in Response to DEX

**Figure 5:**
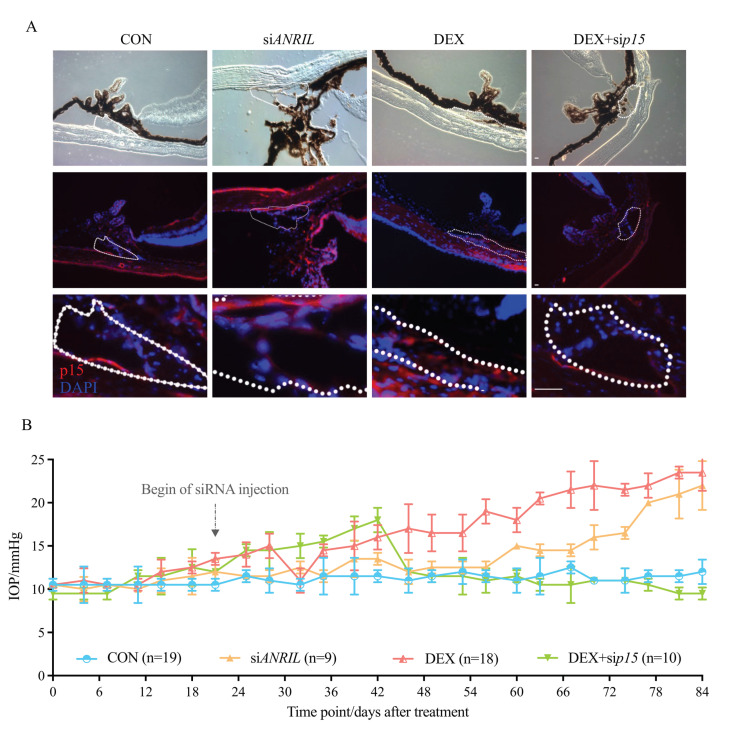
**ANRIL/p15 promotes SIG in mice models.** (**A**) The expression of p15 protein accumulated in mice anterior segment samples after exposure to DEX. p15 knockdown decreased p15 intraocular protein amount. The p15 level in the DEX + sip15 group was comparable to the control mice, suggested by IF. TM region is outlined with white dashes. *n* = 5 per group. (**B**) The IOP significantly increased in DEX-treated mice, starting from 15.3 mmHg at 4 weeks and continually increasing to 23.4 mmHg at 12 weeks. Intraocular ANRIL depletion presented a consistent increased IOP pattern (IOP = 22.1 ± 0.89 mmHg at 12 weeks, compared with control mice, *p* < 0.05). When p15 was knocked-down, IOP elevated in the very beginning period with DEX exposure (0–6 weeks) and then continually recovered to a normal level. Arrow indicates the starting of intracameral siRNA delivery on Day 21. Notes: CON = control; siANRIL = ANRIL knock-down; DEX = dexamethasone; sip15 = p15 knock-down. Scale bar = 10 μm.

Based on these findings, we planned to verify the function of ANRIL/p15 signaling in mice TM in response to DEX. To this end, we introduced a knockdown of ANRIL or p15 in mice eyes. Repeated delivery of intraocular siRNAs ensured the stable depletion of ANRIL or p15 in mice eyes, as measured by immunofluorescence (IF) labeling in mouse anterior segment slices (Figure 5A). The upregulation of p15 in the DEX group was shown in the IF images. The increased p15 expression was mainly localized in the TM region (outlined with white dashes in Figure 5A).

Meanwhile, we witnessed a significant increase of IOP in DEX group, which is the most direct phenotype to infer the TM dysfunction (Figure 5B). As shown in Figure 1A, and again in Figure 5B for comparison, there was a significant increase in IOP in the DEX group, which was elevated from 12.5 to 15.3 mmHg after 4 weeks of treatment, and continually increased to 23.4 ± 1.04 mmHg by the end of Week 12.

Intraocular ANRIL depletion mice without DEX treatment presented a similar pattern of IOP increase (IOP = 22.1 ± 0.89 mmHg in siANRIL group at Week 12, compared with control group *p* < 0.05). Although the extent of IOP increase did not show a statistical significance of 0.5 at 12 weeks between DEX and siANRIL groups, the IOP elevation in siANRIL mice acquired a flatter growth rate compared with DEX group.

When p15 was knocked-down, IOP elevated from 11.6 to 18.1 mmHg after 6 weeks of DEX exposure. However, starting at Week 6, the IOP in DEX + sip15 group gradually recovered to normal levels. At the end of Week 12, IOP DEX + sip15 group (13.1 ± 0.66 mmHg) showed no statistical significance compared with control group (*p* = 0.139). This result suggests the protective effects of p15 depletion against continuous DEX exposure (Figure 5A,B).

### 2.6. ANRIL/p15 Signaling Promotes SIG via TM Cell Senescence

As shown above, ANRIL/p15 signaling promotes IOP elevation in response to DEX in vivo, and its essential role in regulating senescence has been established in cultured TM cells. Based on this evidence, we aimed to confirm the role ANRIL/p15 in mice eyes exposed to DEX. Therefore, anterior segment tissues and TM tissues from different mice groups were collected (details in Methods).

**Figure 6:**
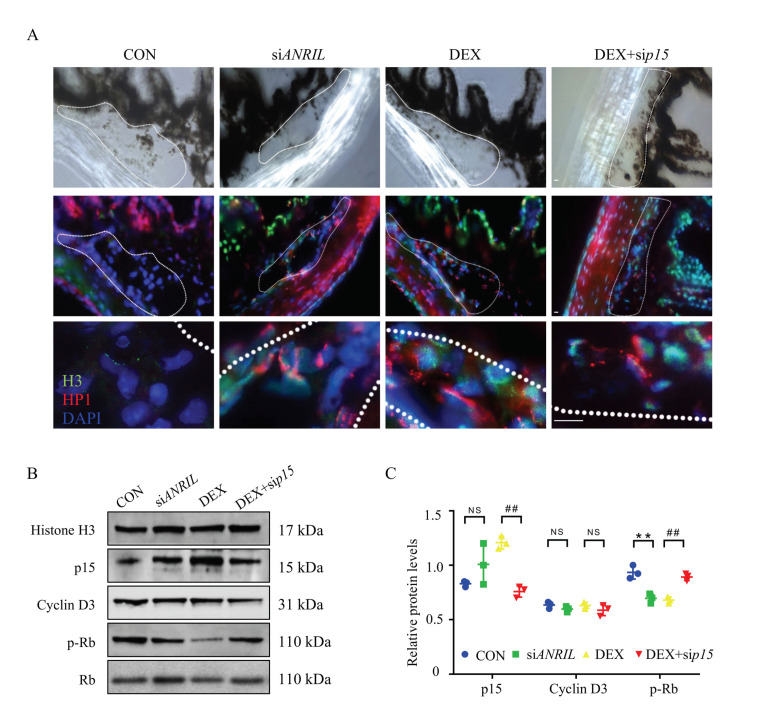
**ANRIL/p15 promotes SIG via TM cell senescence.** (**A**) TM tissues were collected from different mice groups and labeled with SAHF. The cellular senescence rate in TM tissue increased with evident expression of H3 (green) and HP1 (red) in DEX and siANRIL groups. When p15 was depleted, cellular senescence rate was decreased in response to DEX exposure. *n* = 5 per group. (**B**) In isolated mouse TM tissue, the expression of p15 protein and Cyclin D3 protein was measured in nuclear protein fractions, while Histone H3 served as internal control. The phosphorylated Rb protein was checked in cytoplasm fraction with total Rb protein as control. *n* = 6 per group. (**C**) Quantification of (**B**). Notes: CON = control; siANRIL = ANRIL knock-down; DEX = dexamethasone; sip15 = p15 knock down. Scale bar = 10 μm; ** *p* < 0.01 compared with vehicle; ^##^ *p* < 0.01 compared with scramble RNA; NS = no significant difference.

Senescence-associated heterochromatin foci (SAHF) staining was used to label senescent cells in anterior segment slices. Cellular senescence was visualized with H3K9Me2 (H3) and HP-1α (HP1) antibody, as previously explained [33]. As shown in Figure 6A, an increased senescence rate was exhibited with evident expression of H3 (green) and HP1 (red) in response to DEX in anterior segment slices. ANRIL knock-down had a similar effect as DEX with accumulated H3 and HP1 signals in anterior segment slices compared with control mice; the increased senescent cells mostly resided in the TM region. When p15 was depleted, cell senescence rate was decreased even exposed to DEX with less H3 and HP1 (Figure 6A) in TM region, which further validated the potential role of ANRIL/p15 signaling in regulating TM cellular senescence.

TM samples were carefully isolated for protein analyses. As indicated by immunoblot, the protein level of p15 was largely enriched in the TM tissue of DEX and siANRIL mice. The downstream effector, phosphorylated Rb protein, was reduced in DEX and siANRIL group. Meanwhile, p15 knock-down demonstrated protective effect against DEX exposure by regain the phosphorylation of Rb protein in mouse TM (Figure 6B,C). These results were consistent with what we saw in cultured TM cells.

Together, our results suggested that ANRIL/p15 regulated SIG progression through promoting TM cellular senescence in response to steroid both in cultured cells and in mice models.

### 2.7. ANRIL/p15 Signaling Regulated TM Stiffness in HUMAN Samples

**Figure 7:**
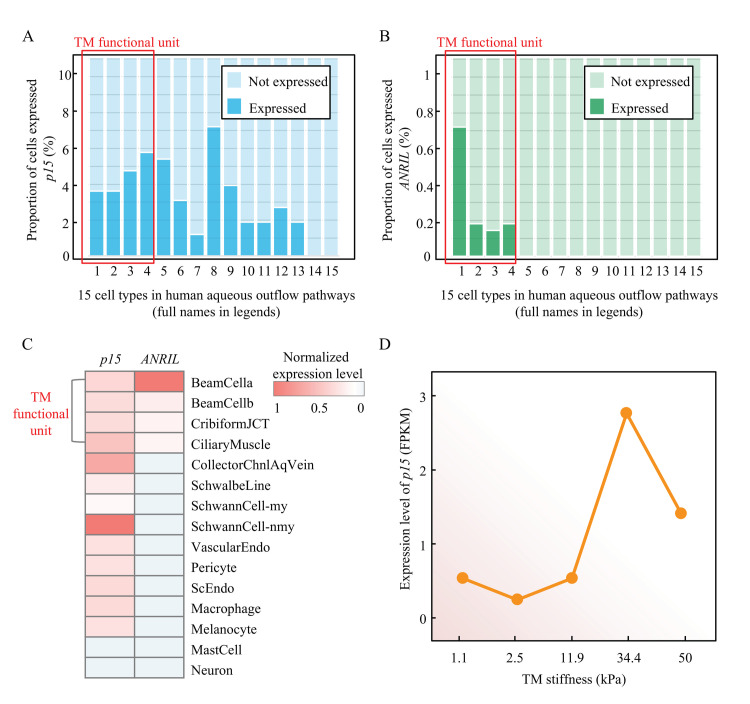
**The ANRIL/p15 expression in human TM cells and its relationship with TM stiffness.** (**A**) The proportion of p15-expressed cells in each cell type in human aqueous outflow pathways. From left to right: **1** beam cells A, **2** beam cells B, **3** juxtacanalicular tissue cells, **4** ciliary muscle cells, **5** collector channels cells, **6** Schwalbe line cells, **7** myelinating Schwann cells, **8** non-myelinating Schwann cells, **9** vascular endothelium cells, **10** pericyte cells, **11** Schlemm canal endothelium cells, **12** macrophages, **13** melanocytes, **14** mast cells, **15** neurons. (**B**) The proportion of ANRIL-expressed cells in each cell type in human aqueous outflow pathways (cell-type order is the same as in panel A). (**C**) The normalized expression level of p15 and ANRIL in each cell type are present in heatmap (blue shallow indicates no expression). (**D**) The expression level (RPKM) of p15 in different TM stiffness are presented.

To validate the role of ANRIL/p15 signaling in human TM, we analyzed the cell atlas of aqueous humor outflow pathways in normal human eyes from Zyl’s study [34] (details in Methods). The results showed that p15 is widely expressed in most of the cell types in aqueous humor outflow pathways, including 4 cell types in TM functional unit (beam cells type A and B, juxtacanalicular tissue cells, and ciliary muscle cells) (Figure 7A). However, the expression of ANRIL is cell-type-specific and enriched in the TM functional unit (Figure 7B). Additionally, the co-expression relationship between p15 and ANRIL are supported by the heatmap (Figure 7C). This evidence supported that the ANRIL/p15 expression is spatially enriched in cells of TM functional unit.

Although detailed mechanism of TM dysfunction resulting in increased resistance remains nascent, a recent study showed the TM is ~20 fold stiffer in glaucoma, suggesting a prominent role of TM mechanobiology [35]. We then analyzed the mRNA expression profiling of human TM cells [36] with definite degrees of stiffness, which is a direct indicator to infer TM dysfunction. As presented in Figure 7D, p15 expression retained a low level in a normal stiffness range (1.1 to 11.9 kPa). With the stiffness increasing into an abnormal range (>11.9 kPa), the p15 expression present a typical upward trend as a proliferative response. It also presented a typical decompensation stage after 34.1 kPa stiffness. This evidence showed a clear association between p15 expression and TM stiffness.

### 2.8. Evidence of ANRIL Underlying Glaucoma Genetic Susceptibility

**Figure 8:**
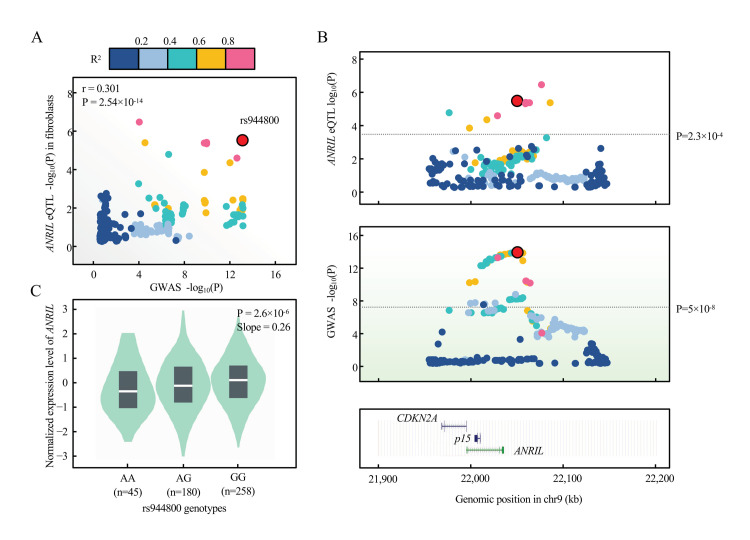
**Colocalization between glaucoma risk variant rs944800 and expression of ANRIL in GTEx.** (**A**) eQTL signal in GTEx v8 fibroblast cells (*n* = 483) for ANRIL colocalizes with that of glaucoma GWAS on the lead variant, rs944800 by coloc (PPH_4_ = 0.688). Pearson correlation is shown between log-transformed *p* values of eQTL (*y*-axis) and GWAS (*x*-axis). Variants are color-coded based on the LD *R*^2^ (1000 Genomes, EUR, phase 3) with the candidate variant (red dot, rs944800). (**B**) Regional association plots of eQTL (blue shadow) and GWAS (green shadow) within +/− 100 kb of rs944800 are presented. The horizontal line indicates genome-wide significant *p*-value for GWAS (5 × 10^−8^) and genome-wide empirical *p*-value threshold for eQTL of ANRIL (2.3 × 10^−4^). UCSC tracks of gene and annotation are displayed as the full mode in this region. (**C**) eQTL violin plots were directly adopted from GTEx v8 data through GTEx portal (gtexportal.org). *p*-value and slope (relative to G allele in rs944800, which is the glaucoma protective allele) were derived from linear regression with no multiple-testing correction.

We explore the possibility that ANRIL can contribute to the glaucoma genetic susceptibility. We first collected 17 SNPs that associated with glaucoma with genome-wide significance, *p* <  5 × 10^−8^, from the NHGRI GWAS Catalog [37] (www.ebi.ac.uk/gwas, accessed 16 April 2021) in the genomic region of ANRIL/p15 (Table 1). When overlaid with *cis*-eQTL of ANRIL from GTEx v8 with empirical genome-wide significance, we observed 2 SNPs (rs944800 and rs523096) that contribute to the expression level of ANRIL (Table 1). We prioritized rs944800 as the candidate variant given that TM cells manifested fibroblast-like phenotype in glaucoma etiology [38,39]. We then performed formal colocalization using GTEx eQTL and GWAS summary statistics of rs944800 (Methods) and our results supported that the expression of ANRIL is colocalized with rs944800 (PPH_4_ = 68.8%) (Figure 8A,B), with risk allele G associated with the decreased expression of *ANRIL* (Figure 8C). This evidence supported that ANRIL is a candidate gene underlying the genetic susceptibility of glaucoma.

## 3. Discussion

Our results supported that reduced ANRIL and increased p15 expression was responsible for TM cell senescence in SIG. When ANRIL was depleted by siRNA, we witnessed similar senescence phenotypes both in cultured TM cells and mice models. Suppression of p15 helps TM cells combat steroid-induced lesions. Steroids achieved effective regulation of cellular senescence by adjusting the expression level of ANRIL/p15 in TM cells.

It should be noted that the promoting role of p15 in cellular senescence has been reported in pancreatic and hepatocellular cancers [40,41]. The expression of p15 was highly upregulated in these tumor tissues to facilitate G1 arrest of tumor cells and cellular senescence. ANRIL has also been implicated in various human diseases by regulating cellular senescence. As reported, the silencing of ANRIL reduces proliferation in fibroblasts and vascular smooth muscle cells [42], which might be the result of premature senescence. This evidence is consistent with our findings on reciprocal role of ANRIL and p15 in cellular senescence of TM cells in SIG.

Although depleting ANRIL level in TM cells introduced similar phenotypic change and functional lesion as steroid, the less percentage of cells blocked in G1 suggested that the detrimental effect in siANRIL group was milder than steroid treatment (Figure 4B–D). In mice, ANRIL knock-down introduced a slower increase of IOP (Figure 5A) and less cellular senescence (Figure 6A–C). This evidence did not challenge the essential role of ANRIL in the pathogenesis of SIG, but warrants further investigation of the ANRIL/p15 independent pathway in SIG.

In addition to SIG, cellular senescence was reported to be essential for POAG and other types of glaucoma. For instance, a risk variant in SIX6 (His141Asn) was found to increase POAG susceptibility by increasing p16 transcription and retinal ganglion cell senescence [43]. An independent study also reported that serine/threonine kinase TANK-binding protein 1 is upregulated upon IOP increase, which induces cellular senescence in glaucoma patients [44]. This above evidence suggests the potential role of cellular senescence in different types of glaucoma.

Notably, it remains unclear whether SIG damage can be mainly attributed to the production of TGF-β. TGF-β has been shown to induce or accelerate senescence and senescence-associated features in various cell types, including fibroblasts, bronchial epithelial cells, and cancer cells [45,46] by inducing cyclin-dependent kinase inhibitors p15, p21, and p27 [47,48]. This evidence introduces the possibility that ANRIL/p15 pathway may be a secondary response regulated by production of TGF-β when exposing to steroids. It would be interesting to disentangle these complexities in SIG in the future.

It should be noted that the genetic component of glaucoma (rs944800, risk allele G) was colocalized with decreased expression of ANRIL in fibroblast cells. Given that TM cells manifested fibroblast-like phenotype in glaucoma etiology [38,39], we hypothesized that individuals with allele G in rs944800 may be susceptible to steroid, which results in decreased expression of ANRIL in TM cells and subsequently functional changes in cell senescence and TM dysfunction. Considering that GWAS susceptibility is not specific to SIG but to the general population, it will be interesting to test our hypothesis in other glaucoma-relevant exposures (e.g., stress) in future studies.

As indicated by clinical guidelines [49], steroids discontinuation is the first step to manage SIG. Nevertheless, discontinuing steroids will significantly increase the risk of recrudescence among patients with autoimmune or inflammatory diseases. Hence, anti-glaucoma medications and surgery are usually required for SIG patients but confers poor visual prognosis due to the limited efficiency [50]. Ophthalmologists and researchers are in urgent need to find solution to eliminate the ocular side effect of steroid treatment. As a potential alternative, the protective role of regulating ANRIL/p15 pathway can be implicated in clinical management of SIG to eliminate the ocular side effect of steroid. Notably, the efficiency of a novel ocular delivery nanosystem has been proved in managing retinal diseases, which hold great promise for translating our findings into clinical practice [51].

Overall, it is essential to characterize the mechanisms and functions of cellular senescence to achieve optimal therapies. Despite the aforementioned questions, this study has the potential to improve clinical management for SIG patients from the aspects of TM cell senescence. Our results already exhibited the protective effect of anti-senescence treatment with p15 depletion in both cultured cells and mice models with SIG. The broad implication of the current research could be achieved for other cellular senescence-related diseases.

## 4. Materials and Methods

### 4.1. Establishment of the Steroid Induced Glaucoma (SIG) Model

Six-week-old C57BL/6J mice were purchased from the Guangdong Medical Laboratory Animal Center. The present study complied with relevant legislation from the Institutional Animal Care and Use Committee of the Zhongshan Ophthalmic Center at Sun Yat-Sen University (No. 2018-014).

We included 100 mice in this study, which were divided into 4 groups: 20 control mice received vehicle (PBS) eye drops, 20 mice received intraocular siANRIL delivery, 30 mice received topical DEX treatment, and 30 mice received both topical DEX and intraocular sip15.

### 4.2. Establishment of the Steroid Induced Glaucoma (SIG) Model

C57BL/6J mice were given topical 0.1% DEX (Sigma-Aldrich, St. Louis, MO, USA) or sterile PBS eye drops (vehicle) 3 times daily, starting at the age of 8 weeks [52]. Every day, the first dose was administered between 9:00 and 10:00 a.m., the second dose was administered between 1:00 and 2:00 p.m., and the third dose was administered between 6:00 and 7:00 p.m. A small eye drop (∼20 μL) was applied to both eyes. Mice were gently held for 30–40 s after drop administration to ensure effective penetration of the eye drops into the anterior chamber, and then released back into their cages. The DEX topical treatments were continued for a total of 12 weeks. Mice with topical PBS treatment served as controls.

### 4.3. Intraocular Pressure (IOP) Measurement

The IOP of mice was measured by a skilled technician, every three days [53]. In brief, mice were anesthetized with 2.5% isoflurane plus 100% oxygen for no longer than 2 min. In both eyes, IOP was measured using an ICare Rebound Tonometer (Tiolat, Oy, Helsinki, Finland). IOP was recorded between 10:00 a.m. and 2:00 p.m.

In the topical DEX-treated group, only mice hat developed significant IOP increase (>21 mmHg) were considered SIG mice. Mice without a response to topical DEX, IOP recovered to baseline during DEX treatment, or present severe ocular (such as edema) or global lesions were excluded from the dataset.

### 4.4. Intracameral Delivery of siRNA

At week 3, the 60 DEX-treated mice were divided into 2 groups: one received topical DEX (DEX group, *n* = 30) and the other received both intracameral siRNA and topical DEX treatment (DEX + sip15 group, *n* =30) for another 9 weeks. Meanwhile, the ANRIL knock-down group received intraocular delivery of ANRIL siRNA without DEX treatment (siANRIL group).

The in vivo siRNA reagent (Ribobio, Guangzhou, CN) was chemically modified and complexed before administrating to ensure the efficiency. A total of 20 μM siRNA (in 1 μL) was used in the intracameral injection. The mice were anesthetized with 2.5% isoflurane plus 100% oxygen. Before operation, mice corneas were topically anesthetized with 0.5% alcaine eye drops. A microinjection syringe (80308, 30 gauge, Hamilton, Reno, NV, USA) was loaded with 1 μL of siRNA complex or vehicle solution without any air bubbles. Under a stereoscopic microscope (M844; Carl Zeiss Meditec, Dublin, CA, USA), a needle was inserted into the anterior chamber of the eye through the limbus in anterior chamber (between cornea and iris), without collapsing the anterior chamber or damage Descemet’s membrane. Slowly the solution was injected into the anterior chamber. The needle was gently withdrawn and no aqueous humor leakage or bleeding was verified. Tobramycin ointment was applied to prevent bacterial infection.

The in vivo siRNA reagent was delivered into the anterior chamber weekly to ensure the intraocular knock-down efficiency by a well-trained and qualified technician. To eliminate the chance of damage or inflammation, we carefully divided the limbus to 12 equal parts, and changed the injection site in a clockwise direction.

Five eyes presented mild to moderate corneal scarring or edema (*n* = 2 in siANRIL group, *n* = 1 in DEX group, *n* = 2 in DEX + sip15 group) and were excluded from microarray, morphology, histology, or IOP analysis, and only included in PCR or WB assay. The eyes that developed severe corneal scarring, neovascularization, edema, or other abnormalities were excluded from the experiment.

### 4.5. Mouse Anterior Segment Isolation

Mice were sacrificed at the end of 12 weeks of treatment, and intracardiac perfusion was conducted with 4% paraformaldehyde (Sigma, USA. Detailed procedures in ref. [54]). Mouse eyes were enucleated after perfusion.

Anterior segments were dissected out carefully under a surgical microscope by an experienced ophthalmologist. The eyes were excised at 0.5 mm posterior to the equator after removing extraocular structures. Retina, choroid, vitreous, and lens were gently removed [55]. The remaining anterior segment was rinsed in PBS followed by 4% paraformaldehyde saline on gentle rotor for 4 h at room temperature. Fixed anterior segment was embedded in optimal cutting temperature compound and kept in −80 °C overnight before sectioning. Samples were sectioned along coronal planes at 10 μm thickness for staining. Mouse anterior segments were used in IF staining and SAHF labeling.

### 4.6. Mouse TM Tissues Isolation

To obtain TM tissues, the anterior portion (as previously isolated) was sliced into four radial wedges. With the TM side facing up, vertical cuts were made with a scalpel down from the TM toward the sclera. Two cuts were made along the anterior (0.5 mm posterior to Schwalbe’s line) and posterior margins (0.5 mm anterior to the scleral spur) of TM [56]. The sclera was removed as much as possible under a microscope without disturbing the TM tissue strip (semi-transparent with some pigmentation). The full TM samples from separated eyes were used in the microarray profiling (*n* = 3 per group), flow cytometry analysis (*n* = 5 per group), and paraformaldehyde fixation (*n* = 5 per group). The rest of the TM samples were bisected, one half was pooled for real-time PCR assay, the other half was pooled for Western blot (*n* = 6 per group).

### 4.7. Mouse TM Samples Microarray Profiling

TRIzol Reagent (Invitrogen, USA) was used to extract total RNA from the TM samples. The Agilent Mouse lncRNA+ mRNA Array V1.0 was used for microarray profiling. |fold change| ≥ 2 and a Benjamini–Hochberg corrected *p*-value < 0.05 were used to designate differentially expressed RNAs. Biological meaning was assigned to dysregulated RNAs in response to DEX exposure using Panther biological system analysis.

### 4.8. Fundus and Optical Coherence Tomography (OCT) Examination

Mice were anesthetized with 1% pentobarbital sodium (i.p., 50 mg/kg, Sigma) and had their pupils dilated with topical tropicamide (Alcon, Belgium). Mice were placed on a rodent alignment stage and hypromellose (Methocel, OmniVision AG, Neuhausen, Switzerland) was applied as optical coupling medium for better imaging resolution. To avoid corneal dryness, 0.9% sterile saline was administered topically to cornea throughout the examination procedure.

Fundus examination was conducted using a retinal imaging system (MicrolV, Phoenix, AZ, USA) [57]. CDR was measured by an experienced researcher three times. RNFL thickness was measured using a Heidelberg Spectralis HRA + OCT system (Heidelberg Engineering, Heidelberg, Germany) according to established protocols [58].

### 4.9. Mouse TM Cells Maintenance and Treatment

The mouse TM cells were obtained from Procell Life Science & Technology Co., Ltd. (Wuhan, China). In brief, TM tissue was micro-dissected from C57BL/6 mice aged between 2–3 weeks old, using sterile precautions. Isolated TM tissue were then placed on a gelatin-coated 6-well plate to facilitate migration of TM cells. Cell cultures were maintained in complete mouse TM cell medium (Procell, Wuhan, China), which contained 5% (*v*/*v*) heat-inactivated fetal bovine serum, 1% TM cell growth supplement, and 1% penicillin/streptomycin. TM cell cultures were kept under 5% CO_2_ and 95% air condition without media changes for 2 weeks. When the tissue adhered to the plate, the media was replaced with fresh media every 2 days. Once the primary culture reached confluency in the 6-well plate, the cells were gently lifted and transferred to a gelatin-coated T-25 flask to allow the primary culture to expand [56]. Once the cell confluency reached 70%, it was ready to use in research and recognized as passage 1. All experiments were performed on cells when they reached about 70–80% confluency within 7 passages.

Two independent lots of commercial mouse TM cells were obtained and used in this study, which ensured the representativity of the results generated using mouse TM cells. To confirm the identification of this commercial cell line, expression of Neuron-specific enolase (NSE) was validated with immunohistochemistry (Figure S1A) [56,59]. In addition, mouse TM cells were treated with 0.1 μM DEX for 3–4 days [60]. An increased expression of myocilin (a marker for TM cell identity) was reported after DEX treatment (Figure S1B).

### 4.10. DEX Treatment in TM Cells

Mouse TM cells were plated at 40–50% confluency for DEX treatment. TM cells were treated with 0.1 μM DEX (Sigma-Aldrich, St. Louis, MO, USA) for 3 days. Cell cultures remained subconfluent during the 3-day treatment. Two independents lots of mouse TM cells were used as biological replicates.

### 4.11. Gene Knockdown in TM Cells

TM cells were plated around 70% confluency for gene knock-down. The siRNA and the scramble were designed and synthesized by the Ribobio Company (Guangzhou, China). The 10 μg/mL siRNAs were complexed in transfection buffer and reagent complexes (Ribobio). After incubation for 30 min, the siRNA mixtures were added dropwise to the culture medium. 48 hours’ transfection was conducted to maximize the knockdown efficiency.

### 4.12. Luciferase Report Assay

TM cells were grown in 96-well plates and attained a confluency of roughly 70% before the experiment. To examine the interaction between ANRIL and p15, mouse p15 (NC_000070.6) gene was cloned upstream of the luciferase reporter. Applied Biological Materials Inc. provided the mouse ANRIL cDNA plasmid (Richmond, Canada). The ANRIL siRNA and the scramble were designed and synthesized by the Ribobio Company (Guangzhou, China). TM cells were co-transfected with luciferase reporter plasmid and ANRIL plasmid or ANRIL siRNA using lipofectamine^TM^ 3000 (Invitrogen, Thermo Fisher, Waltham, MA, USA) according to the manufacturer’s instructions. Negative controls included PcDNA vectors or scramble siRNA.

Cells were lysed 24 h after transfection with 100 μL passive lysis buffer (Dual Luciferase Reporter Assay kit, Promega) into each well. For the luciferase reporter test, 20 μL of cell lysate were employed. Luminescence was quantified in 10 min. A quality control was performed using stable production of the reporter construct, pp15-Luc, or the pGL-3 basic luciferase vector (Promega, Madison, WI, USA). To standardize transfection efficiency, the renilla reporter (RLuc, Promega) plasmid was employed.

### 4.13. Cell Cycle Analysis

Propidium iodide (PI) staining was used to examine the cell cycle distribution of TM cells. Briefly, TM samples were minced into 3 to 4 mm pieces with scissors. TM pieces were added into 4 mg/mL collagenase I (Thermo Fisher Scientific, Waltham, MA, USA) solution (in PBS) and incubated at 37 °C for 2–4 h with gentle agitation until no visible chunks remained. After digestion, cell suspension was spin down at 1000 rpm at 4 °C. Supernatant was removed, and the cell pellet was resuspended in cold PBS and filtered through a 70-μm strainer to prepare single cell suspension. The 1 × 10^6^ TM cells were collected and permeabilized with 70% ethanol, then treated with 200 mg/mL RNase A (Sigma) and 50 mg/mL PI (Sigma) for 10 min before analysis [61]. For cultured TM cells, the same protocol was followed upon obtaining the cell suspension.

Single-cell populations were gated and the percentage of cells in different stages of the cell cycle was determined using Flowjo (Treestar, Inc., Ashland, OR, USA) implementing the Watson (pragmatic) model.

### 4.14. Immunofluorescence (IF) Staining

TM cells were plated on chamber slides (ibidi, Fitchburg, WI, USA). As previously indicated, mouse anterior segment slices were obtained. Slides were fixed with 4% paraformaldehyde, permeabilized with 0.2% Triton X-100, and blocked with 1% bovine serum albumin (BSA).

The samples were incubated with primary antibodies at 4 °C overnight, then for 1 h at room temperature with a species-compatible secondary antibody. The sources and dilutions of antibodies are listed in Table 2. Cell nuclei were stained with 50 ng/mL 4′,6-diamidino-2-phenylindole (DAPI, CST) for 5 min. Images were captured by a Zeiss LSM 510 confocal laser scanning microscope and processed by Adobe Photoshop CS8. In mouse anterior segment slices, the TM region was outlined with white dashes (Figure 5A and Figure 6A) according to the morphology [56].

### 4.15. Immunohistochemistry Labeling

TM cells were seeded on chamber slides (ibidi, Fitchburg, WI, USA) at roughly 70% confluency. Slides were Fixed with 4% paraformaldehyde, permeabilized with 0.2% Triton X-100, and blocked with 1% BSA. Then, the samples were incubated with anti-NSE antibody (Abcam, ab79757) at 4 °C overnight, followed with anti-rabbit IgG, AP-linked antibody (CST, #7054) for 1 h at room temperature. After removing unspecific binding with wash buffer twice, slides were incubated with alkaline phosphatase substrate working solution (VECTOR, Vector^®^ Blue) for 20–30 min in the dark. Images were collected with a Zeiss Episcope and processed by Adobe Photoshop CS8.

### 4.16. Nuclear Extracts

Cultured TM cells or isolated TM tissues were gently resuspended in 500 μL cytoplasmic extract buffer (10 mM HEPES, 60 mM KCl, 1 mM EDTA, 0.075% (*v*/*v*) NP40, 1 mM DTT and 1 mM PMSF, adjusted to pH 7.6) and incubated on ice for 30 min. Mixed solution were then centrifuged at 14,000 rpm for 1 min at 4 °C to obtain the nuclear pellets. Nuclear pellets were resuspended in 100–200 μL nuclear extract buffer (20 mM Tris Cl, 420 mM NaCl, 1.5 mM MgCl_2_, 0.2 mM EDTA, 1 mM PMSF and 25% (*v*/*v*) glycerol, adjusted to pH 8.0) for 30 min on ice with vortex. The extracts were centrifuged at 14,000 rpm for 15 min at 4 °C, and the supernatant was collected for further analyses.

### 4.17. Real-Time PCR

TRIzol Reagent was used to extract total RNA from both TM samples and cultured cells (Invitrogen). The cDNA was synthesized using the PrimeScript RT Master Mix (TaKaRa, Kusatsu, Japan). The primers are listed in Table 3. Quantitative analysis was performed using real-time PCR using the SYBR Advantage qPCR Premix Master Mix (TaKaRa) according to a standard protocol. The cycle threshold (Ct) value of ANRIL and p15 was measured and normalized to β-actin. The comparative Ct method was used to evaluate the expression levels.

### 4.18. Western Blot

Total protein was isolated from TM cells and tissues with RIPA lysis buffer (Beyotime, Shanghai, China). Nucleus protein was isolated from nucleus extracts. Protein samples were electrophoresed on 10% polyacrylamide gels according to a standard procedure. The expression of target proteins was normalized to histone H3 using ImageJ Software. The primary antibodies and dilutions are presented in Table 2.

### 4.19. Bioinformatic Analyses of Human TM Expression Profile

A total of two datasets under accession numbers GSE146188 [34] and GSE123100 [36] were downloaded from the Gene Expression Omnibus database (http://www.ncbi.nlm.nih.gov/geo/ (accessed on 1 July 2020). The GSE146188 is analyzed in the Broad Institute’s Single Cell Portal (https://singlecell.broadinstitute.org/single_cell/study/SCP780 (accessed on 1 July 2020) to compute the expression level of mRNA and lncRNA (Illumina HiSeq 2500). The p15 expression (RPKM) of TM cells in different-level stiffness is analyzed from GSE123100 (GSE123100_cell_expressed_gene_RPKM.txt.gz) and the ANRIL expression is not available in this dataset.

### 4.20. Colocalization between GWAS and eQTL Signals

Given the linkage disequilibrium (LD) discrepancy between the GTEx v8 (~85% European) [54] and Asian GWAS summary statistics of rs944800 [62], we performed the colocalization analysis using a LD-independent Bayesian framework Coloc [63]. We defined the flanking regions to 200 kilobase (kb) on either side of lead GWAS variant rs944800. The PPH_4_ (posterior probability of both traits being associated with the same causal variant) was calculated, and those loci with PPH_4_ > 0.6 were defined as the GWAS–QTL colocalized events [64].

### 4.21. Statistics and Reproducibility

Unless otherwise stated, the experimental results in the figures were indicative of at least three independent repeats. All data were given as means with a standard deviation of one standard deviation (SD). One-way analysis of variance (ANOVA) was performed, followed by the Bonferroni multiple comparison tests and two-way ANOVA using GraphPad Prism data analysis software (version 7.0; GraphPad Software). Statistical significance was defined as a *p*-value of less than 0.05. The association between RNA expression and clinical parameters was tested using Pearson’s technique.

## Figures and Tables

**Table 1:** GWAS risk variants of glaucoma in the genomic region of ANRIL.

*PMID*	*Rsnum*	*Chr*	*Position*	*Nearest Gene*	*Significant eQTL in ANRIL*
22419738	rs1063192	9	22003368	ANRIL	N/A
22792221	rs523096	9	22019130	ANRIL	*p* = 1.6 × 10^−5^ in prostate
22428042	rs7865618	9	22031006	ANRIL	N/A
22570617 32514122 25861811	rs2157719	9	22033367	ANRIL	N/A
26752265 33627673 29891935	rs1333037	9	22040766	ANRIL	N/A
33627673	rs1412829	9	22043927	ANRIL	N/A
29891935	rs10811645	9	22049657	ANRIL	N/A
29452408	rs944800	9	22050899	ANRIL	*p* = 2.6 × 10^−6^ in fibroblast cells
30054594 29891935 31959993 33627673	rs944801	9	22051671	ANRIL	N/A
30104761 33627673	rs6475604	9	22052735	ANRIL	N/A
33627673	rs7853090	9	22056296	ANRIL	N/A
27623284	rs7866783	9	22056360	ANRIL	N/A
21532571 25173105	rs4977756	9	22068653	ANRIL	N/A

**Table 2:** Information and dilutions of primary antibodies.

	Manufacturer	Protein Size	Dilution
p15 antibody	Abcam	15 kDa	WB (1:1000), IF (1:200)
H3K9Me2 antibody	Abcam	17 kDa	IF (1:200)
HP-1α antibody	Boster	20 kDa	IF (1:100)
Phospho-Rb (Ser807/811) antibody	Abcam	110 kDa	WB (1:500)
Histone H3 antibody	CST	17 kDa	WB (1:2000)
Cyclin D3 antibody	Beyotime	31 kDa	WB (1:1000)

**Table 3:** Real-time PCR primers.

Primer Name	Sequence (5′ to 3′)
β-actin-forward primer	TTCTGCTCTTCGGTTCTGCC
β-actin-forward primer	GCCGTGTAGGTCGAAACAGA
p15-forward primer	GGGACTAGTGGAGAAGGTGC
p15-reverse primer	CATCATCATGACCTGGATCGC
ANRIL-forward primer	GCGCCGGACTAGGACTATTT
ANRIL-reverse primer	GCCAGGACGGAGATCAGATG

## Data Availability

The data that support the findings of this study are available from the corresponding author, Y.Z., upon reasonable request.

## Supplementary Materials

The following supporting information can be downloaded at: https://www.mdpi.com/article/10.3390/cells11091468/s1, Figure S1: Validation of mouse TM cells; Table S1: Mouse IOP information.
