# Supplementary material for: Reciprocal Regulation between lncRNA ANRIL and p15 in Steroid-Induced Glaucoma
Source: Cells. 2022 Apr 27;11(9):1468. doi: 10.3390/cells11091468 (PMC9101924; doi:10.3390/cells11091468)
Supplement: Supplementary file 1 [file cells-11-01468-s001.zip › Supplementary Information.pdf]

Supplementary Information

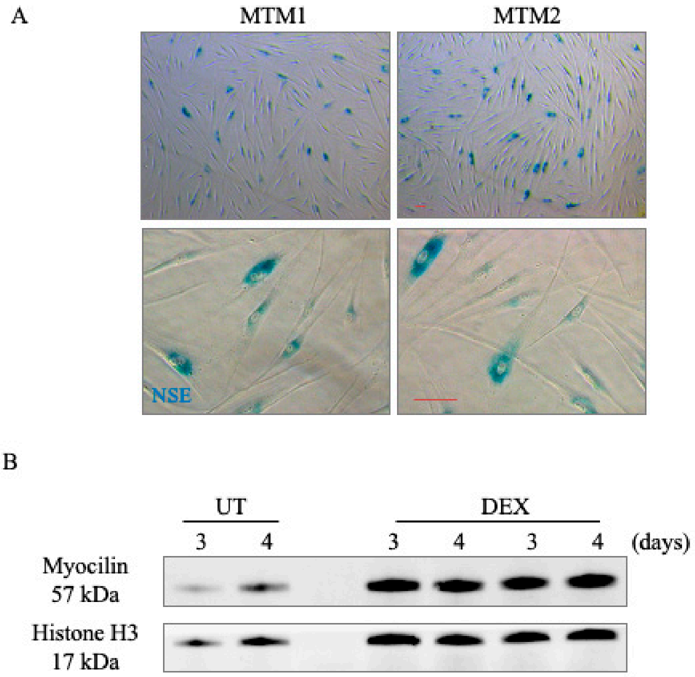

Figure S1. Validation of mouse TM cells

(A) NSE expression was confirmed by immunohistochemistry in two individual vials of mouse TM cells.

(B) Two individual vials of mouse TM cells were treated with 0.1uM dexamethasone for 3-4 days, the myocilin expression in mouse TM cells increased as measured by Western blot.

Notes: n = 2 per group; MTM = mouse trabecular meshwork cells; NSE = Neuron-specific enolase; UT = untreated; DEX = Dexamethasone. Scale bar = 10 μm.
